# Loaded Single-Leg Roman Chair Hold Preferentially Increases Biceps Femoris Activation, Whereas the Nordic Hamstring Exercise Emphasises Semitendinosus Activation in Professional Athletes

**DOI:** 10.3390/medicina62010146

**Published:** 2026-01-12

**Authors:** Gokhan Yagiz, Fuat Yuksel, Cristina Monleón, Hans-Peter Kubis, Gokhan Mehmet Karatay, Serdar Eler, Esedullah Akaras, Nevin Atalay Guzel, Encarnación Liébana

**Affiliations:** 1 Department of Physiotherapy and Rehabilitation, Faculty of Health Sciences, Amasya University, Amasya 05100, Turkey; gokhan.yagiz@amasya.edu.tr; 2 Department of Physical Therapy, Faculty of Health Sciences, Tokyo Metropolitan University, Tokyo 92-0397, Japan; 3 Department of Physiotherapy and Rehabilitation, Faculty of Health Sciences, Ordu University, Ordu 52200, Turkey; 4 Faculty of Physical Education and Sports Sciences, Catholic University of Valencia San Vicente Mártir, 46001 Valencia, Spain; 5 Department of Sport Science, School of Psychology and Sport Science, Bangor University, Bangor LL57 2PZ, UK; 6 Department of Physiotherapy and Rehabilitation, Faculty of Health Sciences, Gazi University, Ankara 06560, Turkey; 7 Faculty of Sport Sciences, Gazi University, Ankara 06560, Turkey; 8 Department of Physiotherapy and Rehabilitation, Faculty of Health Sciences, Erzurum Technical University, Erzurum 25050, Turkey

**Keywords:** biceps femoris, eccentric exercise, electromyography, hamstring strain injuries, injury prevention, Nordic hamstring exercise, Roman chair-hold, semitendinosus

## Abstract

*Background and Objectives*: Hamstring strain injuries (HSIs) are frequent and recurrent in athletes who perform high-speed running. The long head of the biceps femoris (BFlh) is often affected by HSIs. While the Nordic hamstring exercise (NHE) is used for prevention, evidence shows it mainly activates the semitendinosus (ST) instead of the biceps femoris (BF). It was argued that hamstrings may contract isometrically during sprinting’s late swing phase; exercises like the single-leg Roman Chair-Hold (RCH) might better mimic sprinting. Limited electromyographic (EMG) data compare NHE and RCH. This study examined EMG activation of BF and ST during both exercises in athletes. *Materials and Methods*: Thirty-six professional handball players (17 females, 19 males) were randomly assigned to NHE (*n* = 18; mean age 22.1 ± 3.9 years) or RCH (*n* = 18; mean age 22.6 ± 4.9 years). A wireless EMG system recorded dominant leg BFlh and ST activity, normalised to maximal voluntary isometric contraction (MVIC%). NHE participants completed one set of ten repetitions; RCH participants performed three sets of ten repetitions with progressive loads (bodyweight, +10 kg, +20 kg). *Results*: RCH led to a significantly higher mean BFlh activation in the third set with +20 kg weight compared to NHE (72.9% versus 46.5%; *p* < 0.001, *g* = 1.52). BFlh activation steadily increased across RCH sets, coinciding with additional load increments (*p* < 0.001). Conversely, NHE produced greater ST activation than the RCH at the first set, where RCH was performed with only bodyweight (*p* < 0.001). *Conclusions*: NHE primarily activates the ST, while RCH gradually increases BFlh activation, particularly under load. Future research should investigate which exercises are more effective at reducing HSIs to draw more robust conclusions based on the study’s findings.

## 1. Introduction

Hamstring strain injuries (HSIs) frequently occur in sports involving sprinting, spanning from amateur to professional levels [1]. In addition to this, the injury rates have exhibited a gradual upward trend in sports [2]. Furthermore, the recurrence rates of HSIs are also notably high among athletes [2]. Importantly, these recurrent HSIs instances are more severe and result in greater time loss compared to the initial HSIs [3]. Beyond the mentioned reinjury rates, recurrent HSIs may cause enthesopathy, issues with the menisci of the knee, adhesion of the popliteal nerve, motor dysfunction of the ischiatic nerve, abnormal quadriceps strength, and potentially end an athlete’s career [4]. Therefore, scientists have focused on developing an optimal preventive strategy for HSIs before their initial occurrence over the past two decades [5].

Among the hamstring muscles, the biceps femoris long head (BFlh) is the most frequently injured muscle [6]. The late swing phase of high-speed running was identified as the most susceptible period for hamstring injuries [7]. During the late swing phase of running, the hamstring muscles produce eccentric contraction and force to decelerate the tibia and regulate the activity of the quadriceps femoris, which functions as an antagonist to the hamstrings [8]. Among the hamstring muscles, the biceps femoris (BF) attains 109.5% of its original length, surpassing the semitendinosus (ST) at 107.4% and the semimembranosus (SM) at 108.1%, as a result of elongation occurring during the late swing phase of running [9]. HSIs usually happen when muscle fibres fail to withstand excessive force [10]. In this manner, inadequate eccentric contraction of the hamstring muscles during the late swing phase of running was considered the primary cause of HSIs [11].

Subsequently, scientists, medical professionals and sports experts have concentrated on addressing potential deficiencies in the hamstring and have suggested eccentric training methods, including the popular Nordic hamstring exercise (NHE), to prevent future HSI [12,13,14,15]. Recent research utilising electromyography (EMG) [16] and magnetic resonance imaging (MRI) [17,18,19,20] has indicated that the Nordic Hamstring Exercise (NHE) predominantly engages the semitendinosus muscle (ST) rather than the biceps femoris, which is the most commonly injured hamstring muscle owing to its knee-centric function. Furthermore, it is suggested that hip-dominant exercises might elicit greater activation in the biceps femoris relative to knee-dominant exercises.

Conversely, Van Hooren and Bosch [21,22] have recently commenced a discussion regarding the contraction type of the hamstrings during the late swing phase of running, proposing that the hamstrings contract isometrically at this stage. Consequently, Van Hooren and Bosch [21,22] proposed that high-intensity isometric exercises, such as the Single-leg Roman chair hold exercise (RCH), represent a more efficient alternative to eccentric exercises, including the NHE, to prevent HSIs. Following this, McDonald et al. [23] compared the NHE and RCH in terms of their effectiveness in enhancing the strength and endurance of the hamstrings as a whole, as evaluated by the single-leg hamstring bridge test, which had previously been recognised as a risk factor for HSI. Consequently, McDonald et al. [23] stated that the RCH was more effective in enhancing the outcome parameter compared to the NHE. Therefore, competing biomechanical theories exist regarding hamstring fascicle behaviour during the late swing phase in high-speed running [21,22].

In summary, the literature continues to debate whether eccentric training or isometric training offers superior injury prevention for HSI, based on the injury mechanism [21,22,23,24,25,26]. Although the debate continues, there are few studies that compare muscle activation during the NHE and RCH exercises [27]. Therefore, this study aimed to compare hamstring muscle activations during the NHE and RCH training protocols using EMG to identify which exercise targets the BFlh more effectively. The present study does not aim to adjudicate between the superiority of eccentric versus isometric exercises for preventing HSIs; instead, it compares acute neuromuscular activation patterns of key hamstring muscles across the NHE and RCH. The hypotheses of this study are as follows: (1) NHE would induce higher activation in the ST than in the BF muscle, reflecting its knee-dominant focus; (2) RCH would result in greater activation in the BF compared to the ST, due to its emphasis on hip-dominant movements; (3) In the first set, NHE would generate more ST activation than RCH, and RCH would lead higher BF activation than the NHE, where RCH would be performed without external weight as per protocol; (4) In the second and third sets, RCH would lead to increased activation in both BF and ST due to the external weight used during those sets.

## 2. Materials and Methods

### 2.1. Design

This research employed two main designs for each outcome: a repeated measures longitudinal trial for RCH measurements and a quasi-experimental, cross-sectional study for within- and between-group muscle comparisons following the STROBE guidelines [28]. Although crossover designs are common in EMG studies for comparing muscle activation after exercise, this study specifically avoided using such a design to prevent prior exercise from influencing subsequent muscle activation measurements. For the EMG measurement procedures, the Surface Electromyography for Non-invasive Assessment of Muscles (SENIAM) guidelines [29] was followed by the researchers.

Recruitment of the participants and data collection for this study were performed at Gazi University facilities and laboratories in Ankara/Türkiye. This study received ethics approval from the Research Ethics Committee of Erzurum Technical University (Approval No. 02-6). All participants reviewed and signed a written informed consent form prior to engaging in the study, in accordance with the ethical principles outlined in the Declaration of Helsinki for medical research involving human subjects [30].

This research analysed the activation of the medial (ST) and lateral (BF) hamstring muscles during the RB and NHE performances employing a wireless 8-channel EMG system (Noraxon MiniDTS, Noraxon, USA, Inc., Scottsdale, AZ, USA). Prior to the assessments of maximal voluntary isometric contraction (MVIC), participants engaged in a 5 min warm-up on an exercise bicycle. They were advised to pedal at their own pace without adhering to specific intensity guidelines. The participants’ height, body mass, body mass index (BMI), gender, and age were documented prior to the intervention.

### 2.2. Participants

Thirty-six professional handball players from the Turkish Super Leagues participated in this study (17 females and 19 males) under two groups: NHE (*n* = 18, comprising 9 females and 9 males) and RCH (*n* = 18, comprising 8 females and 10 males). All participants read and signed a written informed consent form prior to participating in the study. Participants were advised not to engage in any unfamiliar or vigorous-intensity training 24 h before the measurements [31]. The inclusion criteria were delineated as follows: (a) participants must be professional athletes affiliated with a recognised sports team; (b) participants must not present with an acute injury to the lower extremities; (c) participants must have no history of hamstring injury or traumatic knee injuries, such as anterior cruciate ligament injury. The exclusion criteria were specified as follows: (a) participants must not have experienced recent injuries to the lower extremities; (b) participants must not have a history of hamstring injury or traumatic knee injuries; (c) participants must not be diagnosed with cardiovascular disease; (d) participants must not possess an implanted pacemaker. Prior to the commencement of interventions, the health status of each participant was evaluated using the Physical Activity Readiness Questionnaire (PAR-Q) [32].

### 2.3. Exercise Protocols

The RCH group performed three sets of allocated exercise by following a training protocol of McDonald et al. [23]. due to the different additional weight-bearing quantities in each set. However, the NHE group performed only one set of the exercise, as they repeated the same activity in each set to prevent potential muscle soreness that could impact the player before a competitive game. Participants performed ten repetitions per set in each group. The RCH group was trained with external weight bearing during the second and third sets, in accordance with the protocol of McDonald et al. [23]. A 10 s pause was applied between repetitions, and all of the repetitions of each set were documented for electromyography (EMG) assessment. The concentric phases of the Nordic Hamstring Exercise (NHE) movements were not executed [33]. Additionally, only the single-leg hold and the barbell-lowering segment of the RCH were documented [27]. The exercises were performed in accordance with the instructions provided by Van Hooren et al. [27]. There were three-minute intervals between each set [33].

#### 2.3.1. Nordic Hamstring Exercise

**Figure 1:**
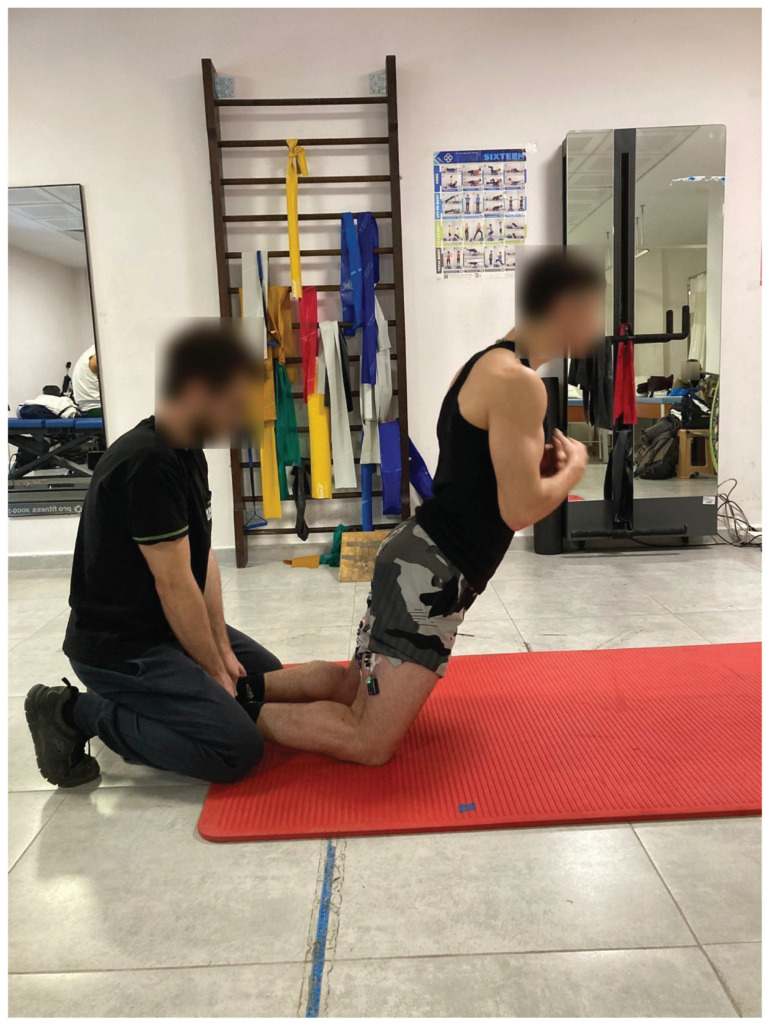
Nordic Hamstring Exercise. A depiction showing a moment from the Nordic hamstring exercise. The participants consented to being identifiable in the image.

The NHE was carried out by the NHE group participants as outlined by Petersen et al. [14]. A partner provided support to the participants’ ankles by applying pressure, ensuring that their lower legs and ankles remained in contact with the ground throughout the movement. Initially, when participants’ knees were bent at a 90° angle, their hips and spines maintained a neutral position (see Figure 1). Subsequently, participants commenced a forward-falling motion and eccentrically engaged their muscles to resist this movement. They were instructed to decelerate their forward fall for as long as possible in order to diminish their velocity. At the conclusion of the movement, participants made contact with the floor using their hands and arms, allowing their chest to touch the ground before promptly returning to the initial position by exerting force against the floor with their hands and arms, thereby minimising the concentric phase of the movement [13]. To maintain a continuous angular velocity, researchers gave instructions to participants as outlined by Burrows et al. [33]. Researchers counted respectively “5 s, 4 s, 3 s, 2 s and 1 s” during the downward phase of the NHE from beginning to end [33]. The participants completed ten NHE repetitions, with 10 s breaks between each repetition and the concentric phase of the movement was not performed [33].

#### 2.3.2. Single-Leg Roman Chair Hold Exercise

**Figure 2:**
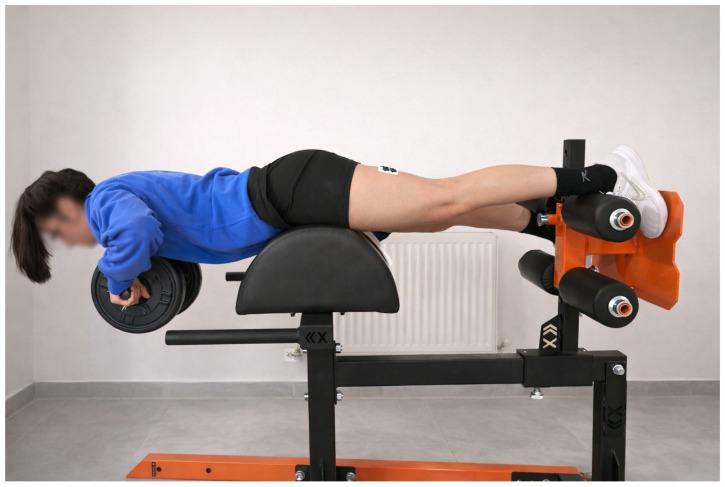
An illustration of the single-leg Roman chair hold exercise is presented during the third set. The individual depicted consented to the publication of her photograph.

A standard glute–hamstring machine (Figure 2) was employed to execute the RCH exercise, which predominantly induces isometric contraction [22]. RCH exercise was completed in accordance with the work published by McDonald and colleagues [23]. Participants adopted a prone position on the machine, with their knees slightly extended. The ankle of the participants’ dominant leg was stabilised beneath the pad of the glute–ham machine. Subsequently, they elevated their trunks until attaining a neutral hip-lumbar position. The contralateral leg was positioned above the pad of the glute–hamstring machine to establish the single-leg condition of the exercise. This exercise was performed on the participants’ dominant legs to ensure comparable results with the NHE, and all measurements were taken from the leg executing the exercise.

### 2.4. EMG Measurement Procedures

During the measurement process, no electronic devices, including cell phones, were in the measurement room. Additionally, the computer’s power supply was disconnected to avoid external electronic interference during electromyography (EMG) measurements. The positions of surface EMG sensors on the BF and ST muscles were chosen based on the SENIAM guidelines [29]. According to the SENIAM guideline, BF refers to the lateral hamstrings, and ST indicates the medial hamstring muscles [29]. The EMG sensor sites were shaved before the warm-up, and the areas were cleaned with an isopropyl alcohol swab to lower skin impedance [34]. The surface EMG sensors were placed at the midpoint between the ischial tuberosity and the lateral epicondyle of the tibia for the BF, and at the midpoint between the ischial tuberosity and the medial epicondyle of the tibia for the ST, aligned parallel to the muscle fibres [29] on both participants’ legs [35]. However, EMG activities were recorded only from the muscles of the participants’ dominant legs.

A wireless EMG system, Noraxon MiniDTS (Noraxon Inc.), was used during data collection. It features a rejection ratio of over 100 dB, a differential input impedance exceeding 100 MOhm, and a sampling rate between 1500 and 3000 Hz per channel [36]). The electrodes were spaced 20 mm apart, with each self-adhesive EMG electrode having a 10 mm diameter [36]. Possible artefacts were visually identified during data collection [36]. The EMG signals were analysed and processed with MR 3.12 software (Noraxon Inc.) [36]. A band-pass filter covering 20–450 Hz was used to process the raw EMG signals [36]. The EMG data were processed with a 100-millisecond moving RMS to smooth the signals [36]. Two MVICs were conducted before the intervention to normalise the EMG data. During these MVICs and ten repetitions of each exercise, EMG activity was recorded for each muscle (BF and ST) during each exercise.

### 2.5. EMG Data Normalisation via MVIC

After the warmup, participants performed two MVICs on a standard medical bed in a prone position with their dominant legs flexed at 45°. An isokinetic dynamometer (Cybex Humac Norm 360, CSMI solutions, Stoughton, MA, USA) measured the MVICs, and EMG signals from the BF and ST muscles of the dominant legs were recorded. Each participant completed two 5 s MVICs for the dominant legs, with a 1 min rest interval between them [33]. The average peak and RMS of surface EMG across two measurements were calculated and used to normalise the mean and peak EMG data from the exercise interventions, expressed as a percentage of MVIC [33] using these formulas:

For mean EMG activity:

$$\frac{\;Mean\;RMS\;during\;the\;exercise\;for\;each\;muscle}{\;Mean\;RMS\;during\;the\;MVIC\;at\;the\;isokinetic\;machine\;for\;each\;muscle}\times 100$$

For maximum EMG activity:

$$\frac{Peak\;RMS\;during\;the\;exercise\;for\;each\;muscle}{\;Peak\;RMS\;during\;the\;MVIC\;at\;the\;isokinetic\;machine\;for\;each\;muscle}\times 100$$

### 2.6. Statistical Analyses

Sample size calculation was conducted utilising G*Power software (version 3.1.9.7) [37] for the F test, with an effect size of 0.5, 80% power, an alpha level of 0.05, and two groups. The total required sample size was established at 34 participants. Nevertheless, 36 participants (comprising 17 females and 19 males) were recruited to ensure adequate statistical power for this study. Statistical analyses were carried out using repeated measures Analysis of Variance (ANOVA) and one-way ANOVA to compare outcome measures between groups and muscles, employing IBM SPSS 29.0 software (IBM Corp, Armonk, NY, USA). Moreover, the Bonferroni Post Hoc test was used to further assess differences between the groups and sets. Furthermore, Hedges’ adjusted *g* effect size was also calculated, which adjusts for potentially biased small sample sizes (*n* < 20), unlike Cohen’s d effect size [38]. The Hedges’ *g* effect size was classified as follows: small (0.2), medium (0.5), or large (0.8) [39].

## 3. Results

There were no significant differences between the RCH and NHE groups regarding the age (22.1 ± 3.9 vs. 22.6 ± 4.9, *p* = 0.735), body mass (76 ± 15.0 vs. 74.7 ± 11.5, *p* = 0.766), body mass index (24.0 ± 2.7 vs. 23.6 ± 2.0, *p* = 0.601) and height (177.1 ± 8.5 vs. 177.6 ± 10.4, *p* = 0.875) (Data S1. pdf). As demonstrated in Table 1, the RCH exercise resulted in a significantly higher mean electromyographic (EMG) activation of the BF muscle in comparison to the NHE, with values of 56.9 ± 16.5% versus 46.5 ± 9.6% of maximum voluntary isometric contraction (MVIC), respectively (*p* = 0.028, *g* = 0.8). During the third set of RCH, BF activation (72.9 ± 22.6%) was notably greater than the activation observed during the first set of NHE (46.5 ± 9.6%) (*p* < 0.001, *g* = 1.52). Additionally, the second set of RCH exhibited a tendency toward higher activation (55.5 ± 17.0%); however, this difference did not achieve statistical significance (*p* = 0.060, *g* = 0.65). Regarding peak electromyography (EMG) readings, the NHE and RCH groups exhibited comparable mean values (63.4 ± 13.1% versus 68.4 ± 20.4% MVIC, *p* = 0.384, *g* = 0.30). Nevertheless, the third set of the RCH group demonstrated a significantly greater biceps femoris (BF) peak activation (86.2 ± 27.6%) compared to the NHE group (63.4 ± 13.1%) (*p* = 0.003, *g* = 1.05). Furthermore, BF activation notably increased across the RCH sets, with statistically significant distinctions observed between Set 1 and Set 2 (*p* < 0.001), Set 1 and Set 3 (*p* < 0.001), as well as Set 2 and Set 3 (*p* < 0.001) (Table 2).

### 3.1. Semitendinosus Activation

According to Table 3, the average of mean EMG activation of the ST did not differ significantly between the NHE and RCH (53.2 ± 13.7% vs. 47.8 ± 20.0% MVIC, *p* = 0.351, *g* = 0.32). However, during the initial set, the NHE elicited substantially higher ST activation (53.2 ± 13.7%) compared to the RCH (34.4 ± 17.4%) (*p* < 0.001, *g* = 1.20).

For peak EMG activity, the NHE exhibited a higher mean activation level (71.0 ± 22.1%) compared to the RCH (56.2 ± 24.4%), approaching statistical significance (*p* = 0.065, *g* = 0.64). The initial set of the NHE demonstrated a significantly greater peak semitendinosus (ST) activation (71.0 ± 22.1%) than RCH (39.7 ± 21.5%) (*p* < 0.001, *g* = 1.43). No significant differences were observed between groups in the second (*p* = 0.068, *g* = 0.63) or third (*p* = 0.790, *g* = 0.09) sets.

Within the RCH group, the mean and peak activations of ST increased progressively across the sets (Table 2). The average electromyography (EMG) values elevated from 34.4 ± 17.4% in the first set to 46.5 ± 20.8% in the second set (*p* < 0.001), and further to 62.5 ± 24.3% in the third set (*p* < 0.001). Similarly, peak EMG values demonstrated an increase from 39.73 ± 21.5% in the first set to 55.6 ± 26.6% in the second set, and subsequently to 73.2 ± 28.2% in the third set, with all differences reaching statistical significance (*p* < 0.001) (Table 2).

### 3.2. Within-Group Comparisons Between Muscles

Within the RCH group (Table 4), the BF exhibited higher levels of activation compared to the semitendinosus (ST) in both mean and peak EMG measurements; however, these differences did not reach statistical significance. The average of mean EMG was 56.9 ± 16.5% for BF and 47.8 ± 20.0% for ST (*p* = 0.146, *g* = 0.50), indicating a moderate effect size despite the lack of significance. Likewise, the average peak EMG values were insignificantly elevated for BF (68.4 ± 20.4%) relative to ST (56.2 ± 24.4%) (*p* = 0.111, *g* = 0.55), also demonstrating a moderate effect size.

Within the NHE group (Table 5), the ST demonstrated greater activation compared to the BF, although these differences did not attain statistical significance. The mean electromyography (EMG) values were 53.2 ± 13.7% for the ST and 46.5 ± 9.6% for the BF (*p* = 0.099, *g* = 0.57), indicating a medium effect size. Similarly, peak EMG activation favoured the ST (71.0 ± 22.1%) over the BF (63.4 ± 13.1%) (*p* = 0.220, *g* = 0.42), reflecting a small-to-medium effect size.

### 3.3. Overview of Muscle Activation Patterns in the Exercises

Overall, NHE elicit higher hamstring activation during the initial set, especially in the ST muscle. RCH shows a steady increase in activation across sets, especially in the BF, due to added weight (+10 kg in the second set, +20 kg in the third). RCH did not initially activate the BF more than NHE, but by the third set, BF activation in RCH surpassed NHE in mean (*p* < 0.001, *g* = 1.52) and peak (*p* = 0.003, *g* = 1.05) EMG. This suggests BF engagement during RCH increases with load, making it effective for BF when performed with added weight over multiple sets. During RCH, the BF consistently showed higher activation than the ST (mean EMG 56.9% vs. 47.8%; *p* = 0.146, *g* = 0.50; peak EMG 68.4% vs. 56.2%; *p* = 0.111, *g* = 0.55), despite not reaching significance. In NHE, the ST tended to be more active than the BF (mean EMG 53.2% vs. 46.5%; *p* = 0.099, *g* = 0.57; peak EMG 70.95% vs. 63.38%; *p* = 0.220, *g* = 0.42). In summary, NHE mainly activates the semitendinosus, and RCH increasingly activates the BF across sets.

## 4. Discussion

The main findings showed that the RCH caused significantly higher average BF activation than the first NHE set, especially in the third set. Conversely, the NHE mainly activated the ST during the initial set. These findings support the idea that each exercise has unique activation patterns, which may be due to their different biomechanical focuses: the NHE predominantly uses the knee, whereas the RCH relies on the hip rather than the knee for movement. Additionally, increasing load in the RCH led to a steady rise in activation for both BF and ST across sets, which could indicate improved neuromuscular recruitment with added external weight.

The study findings indicate that the NHE preferentially targeted the medial hamstrings. The NHE involves controlled forward leaning against gravity with eccentric knee flexion, which resulted in high medial hamstring activity [17,18,19,20]. In addition to the EMG and MRI-based evidence [17,18,19,20], recent studies found ST targeting immediate changes in hamstring muscle stiffness after one NHE session, which agrees with the study highlighting the trend of the higher ST than the BF activation during NHE [40,41].

In contrast, this study’s findings indicate that the RCH primarily activated the lateral hamstring. The RCH maintains a near-isometric contraction at the hip, which primarily targets the lateral hamstrings, which contribute more substantially to hip extension [21,27]. Van Hooren et al. [27] demonstrated through musculoskeletal modelling and ultrasonography that the RCH generates high passive tension and fascicle shortening in the BF, consistent with quasi-isometric contraction patterns. These authors concluded that the RCH may simulate the isometric behaviour of the hamstrings during the late swing phase of sprinting, which challenges the traditional assumption that the hamstrings contract eccentrically during this phase [21,22]. The present findings reinforce this concept, showing that RCH induces higher BF activation under load, thereby providing a potentially sport-specific stimulus for the muscle most susceptible to strain injuries. Nevertheless, the issue remains unresolved and requires further descriptive and prospective research to enhance understanding of injury mechanisms and preventive strategies.

Interestingly, BF activation increased progressively across RCH sets, which coincided with external loads of 10 kg and 20 kg in the second and third sets, respectively. This progressive response may suggest a dose-dependent neuromuscular adaptation to higher hip-extension torque demands. The increase in BF activation across sets in the current study likely reflects cumulative recruitment of high-threshold motor units as fatigue developed, which further emphasises the RCH’s potential suitability for strength-endurance training. McDonald et al. [23] showed that RCH enhanced hamstring strength-endurance, evidenced by better single-leg hamstring bridge performance compared to NHE. Moreover, the RCH’s ability to sustain high activation levels under isometric or quasi-isometric conditions may enhance muscle-tendon stiffness and load tolerance [21,27,42,43,44]. Conversely, the NHE produced significantly greater ST activation in the first set than the RCH, which confirms the knee-dominant, medial hamstring bias reported in previous studies [17,18,19,20]. Although such adaptations enhance overall hamstring strength, they may not optimally target the BF, the muscle most commonly injured during sprinting [6].

Within-group comparisons indicated that, although not statistically significant, the BF consistently exhibited higher mean and peak activations than the ST during RCH, with a moderate effect size. This trend corresponds to the mechanical reasoning and assumption that exercises which involve hip extension may primarily activate the BF [19,27,35]. Similarly, within the NHE group, the ST activation was higher than that of the BF, which again supports previous EMG findings [17,18,19,20]. The activation patterns across exercises suggest that each exercise engages particular hamstring regions and functions, which can potentially be used to guide personalised training plans. Typically, the NHE is performed without external resistance, but adding progressive loads during RCH improves BF engagement, indicating that only the third RCH set, with the heaviest load, produced significantly higher BF activation than NHE in this study. Future studies should clarify whether the hamstrings produce eccentric or isometric contractions during the late swing phase of running and which contraction type-based exercises are more effective for reducing HSIs.

This study may have limitations that affect how its results are interpreted and how widely they can be applied. The study’s focus on a single centre, the relatively small sample size, and the exclusive inclusion of professional handball players may further limit its generalisability to a broader athletic population, including football, rugby, Australian rules football, track and field, and American football athletes. The protocols were not volume- or load-matched because RCH used progressive external loads across three sets, whereas NHE used only one, which could have affected EMG amplitudes due to fatigue or potentiation. Consequently, comparisons between exercises should be made cautiously at the protocol level. A parallel-group design was chosen to minimise carry-over effects from exercise familiarity and repeated bouts, which was important given the time constraints of professional athletes. We acknowledge that between-group designs can introduce inter-individual variability; therefore, our findings are mainly viewed as differences in activation patterns within the context of the specific protocols, rather than as definitive volume-matched comparisons. Because EMG amplitude may be influenced by fatigue, pacing, and intensity, direct comparisons between exercises should be understood as reflecting the protocols tested rather than isolating the pure ‘exercise effect’ under standardised conditions. Additional limitations of this study include the lack of randomisation and blinding, and the measurement of only the dominant limb, which should be addressed in future studies. Surface electromyography (EMG) provides an indirect measure of muscle force and tension, but the amplitude of EMG signals does not always accurately reflect muscle force across various types of contractions or at different movement speeds: For instance, eccentric contractions can produce substantial muscle force despite low EMG amplitude, which complicates interpreting activation data as an indicator of mechanical load [45]. Furthermore, increased movement speeds or intricate joint motions can influence motor unit recruitment, making the link between EMG amplitude and true muscle output more complicated [45]. This methodological limitation is especially relevant for dynamic, multi-joint tasks like the NHE and RB exercises, where joint angles, contraction speeds, and neuromuscular coordination vary during movement. Therefore, although the EMG data in this study offer valuable comparative insights into muscle activity, it should not be regarded as a conclusive measure of exercise intensity or tissue load across different exercises.

Additionally, Surface EMG also faces practical issues. Since surface EMG amplitude indicates neuromuscular activation instead of muscle force or tissue strain, our findings should be viewed as variations in activation strategies across different exercises, rather than direct indications of hamstring force, fascicle strain, or injury prevention effectiveness. Crosstalk from nearby muscles and changes in skin impedance can cause noise, despite steps like shaving and cleaning the skin to reduce these effects. The study did not measure skin resistance quantitatively, which limited its ability to fully account for signal variability. Future studies could enhance accuracy by using intramuscular (needle) EMG for more precise, localised muscle activity recordings, especially in smaller or deeper muscles. Another concern is electrode placement, which followed SENIAM guidelines but was not verified with ultrasound imaging. This might result in misalignment between the sensor and muscle fascicles, particularly in individuals with different anatomy. Employing ultrasound guidance for electrode placement could improve accuracy and decrease EMG signal variability. While the cross-sectional design provides a snapshot of muscle activity during each exercise, it does not offer insights into long-term training effects or injury prevention. Future longitudinal studies are needed to assess whether differences in EMG activation lead to significant physiological or functional improvements related to hamstring strain injuries (HSIs). Future research should investigate whether the hamstrings perform eccentric or isometric contractions during the late swing phase of running and identify which types of contraction exercises are more effective in reducing HSIs, which may provide more insights into the study’s findings.

## 5. Conclusions

The Nordic Hamstring Exercise preferentially elicited higher activation of the semitendinosus, while the single-leg Roman chair hold consistently produced greater biceps femoris activation, especially with external loading and across repeated sets. Because the biceps femoris is the hamstring muscle most often involved in high-speed running injuries, the pronounced activation observed during the hip-dominant Roman chair hold underscores its potential value in hamstring-strengthening programmes. Overall, these findings support the inclusion of both knee-dominant and hip-dominant exercises to achieve more comprehensive hamstring activation in professional athletes.

## Figures and Tables

**Table 1:** Changes in the BF electromyographic activity (MVIC% in mean ± SD) based on the groups.

	NHE (*n* = 18)	RCH (*n* = 18)	*p*-Value	Effect Size (Hedges’ (Adjusted) *g*)
Mean average EMG for BF	46.5 ± 9.6	56.8 * ± 16.5	0.028	0.77
Mean EMG for 1st set for BF	46.52 ± 9.6	42.35 ± 15.2	0.331	0.33
Mean EMG for 2nd set for BF	Not performed, the first set of results was used for comparison (46.52 ± 9.60)	55.45 ± 17.0	0.060	0.65
Mean EMG for 3rd set for BF	Not performed, the first set of results was used for comparison (46.52 ± 9.60)	72.9 ** ± 22.6	<0.001	1.52
Peak average EMG for BF	63.38 ± 13.12	68.4 ± 20.4	0.384	0.30
Peak EMG for 1st set for BF	63.38 ± 13.12	52.0 ± 20.9	0.059	0.65
Peak EMG for 2nd set for BF	Not performed, the first set of results was used for comparison (63.38 ± 13.12)	67.1 ± 20.6	0.522	0.22
Peak EMG for 3rd set for BF	Not performed, the first set of results was used for comparison (63.38 ± 13.12)	86.2 * ± 27.6	0.003	1.05

Abbreviations: BF, biceps femoris; EMG, electromyography; MVIC%, percentage ratio to maximal voluntary isometric contraction; NHE, Nordic Hamstring exercise; RCH, single-leg Roman Chair-Hold exercise; SD, standard deviation; *, significantly higher (*p* < 0.05); **, significantly higher (*p* < 0.001).

**Table 2:** Differences in electromyographic (MVIC% in mean ± SD) activations between sets for each muscle and the significance of the differences for the RCH group.

	Set 1	Set 2	Set 3	*p*-Values
Mean EMG activity of the BF	42.4 ± 15.2	55.5 ± 17.00 **^,**α**^	72.9 ± 22.6 **^,**α**,**β**^	Set 1 vs. Set 2: < 0.001 Set 1 vs. Set 3: < 0.001 Set 2 vs. Set 3: < 0.001
Peak EMG activity of the BF	52.0 ± 20.9	67.1 ± 20.6 *^,**α**^	86.2 ± 27.6 **^,**α**,**β**^	Set 1 vs. Set 2: 0.002 Set 1 vs. Set 3: < 0.001 Set 2 vs. Set 3: < 0.001
Mean EMG activity of the ST	34.4 ± 17.4	46.5 ± 20.8 **^,**α**,**β**^	62.5 ± 24.3 **^,**α**,**β**^	Set 1 vs. Set 2: < 0.001 Set 1 vs. Set 3: < 0.001 Set 2 vs. Set 3: < 0.001
Peak EMG activity of the ST	39.7 ± 21.5	55.6 ± 26.6	73.2 ± 28.2	Set 1 vs. Set 2: < 0.001 Set 1 vs. Set 3: < 0.001 Set 2 vs. Set 3: < 0.001

Abbreviations: BF, biceps femoris; EMG, electromyography; MVIC%, percentage ratio to maximal voluntary isometric contraction; SD, standard deviation; ST, semitendinosus; *, significantly higher (*p* < 0.05); **, significantly higher (*p* < 0.001); ^α^, significantly higher than Set 1; ^β^, significantly higher than Set 2.

**Table 3:** Changes in the ST electromyographic activity (MVIC% in mean ± SD) based on the groups.

	NHE (*n* = 18)	RCH (*n* = 18)	*p*-Value	Effect Size (Hedges’ (Adjusted) *g*)
Mean average EMG for ST	53.2 ± 13.7	47.8 ± 20.0	0.351	0.32
Mean EMG for 1st set for ST	53.2 ** ± 13.7	34.4 ± 17.4	<0.001	1.20
Mean EMG for 2nd set for ST	Not performed, the first set of results was used for comparison (53.20 ± 13.67)	46.5 ± 20.8	0.264	0.38
Mean EMG for 3rd set for ST	Not performed, the first set of results was used for comparison (53.20 ± 13.67)	62.5 ± 24.3	0.166	0.47
Peak average EMG for ST	70.95 ± 22.10	56.2 ± 24.4	0.065	0.64
Peak EMG for 1st set for ST	70.95 ** ± 22.10	39.7 ± 21.5	<0.001	1.43
Peak EMG for 2nd set for ST	Not performed, the first set of results was used for comparison (70.95 ± 22.10)	55.6 ± 26.6	0.068	0.63
Peak EMG for 3rd set for ST	Not performed, the first set of results was used for comparison (70.95 ± 22.10)	73.2 ± 28.2	0.790	0.09

Abbreviations: EMG, electromyography; MVIC%, percentage ratio to maximal voluntary isometric contraction; NHE, Nordic Hamstring exercise; RCH, single-leg Roman Chair-Hold exercise; SD, standard deviation; ST, semitendinosus; **, significantly higher (*p* < 0.001).

**Table 4:** Electromyographic (MVIC% in mean ± SD) changes based on the muscles in the RCH group.

	BF (*n* = 18)	ST (*n* = 18)	*p*-Value	Effect Size (Hedges’ (Adjusted) *g*)
Average of the mean EMG for the three sets	56.9 ± 16.5	47.8 ± 20.0	0.146	0.50
Mean EMG for 1st set	42.4 ± 15.2	34.4 ± 17.4	0.153	0.49
Mean EMG for 2nd set	55.5 ± 17.0	46.5 ± 20.8	0.168	0.47
Mean EMG for 3rd set	72.9 ± 22.6	62.5 ± 24.3	0.195	0.44
Average of the peak EMG for the three sets	68.4 ± 20.4	56.2 ± 24.4	0.111	0.55
Peak EMG for 1st set	52.0 ± 20.9	39.7 ± 21.5	0.091	0.58
Peak EMG for 2nd set	67.1 ± 20.6	55.6 ± 26.6	0.156	0.48
Peak EMG for 3rd set	86.2 ± 27.6	73.2 ± 28.2	0.173	0.46

Abbreviations: BF, biceps femoris; EMG, electromyography; SD, standard deviation; ST, semitendinosus.

**Table 5:** Electromyographic (MVIC% in mean ± SD) changes based on the muscles in the NHE group.

	BF (*n* = 18)	ST (*n* = 18)	*p*-Value	Effect Size (Hedges’ (Adjusted) *g*)
Mean EMG for 1st set	46.5 ± 9.6	53.2 ± 13.7	0.099	0.57
Peak EMG for 1st set	63.4 ± 13.1	71.0 ± 22.1	0.220	0.42

Abbreviations: BF, biceps femoris; EMG, electromyography; MVIC%, percentage ratio to maximal voluntary isometric contraction; SD, standard deviation; ST, semitendinosus.

## Data Availability

All the relevant data are provided in Data S1.pdf.

## Supplementary Materials

The following supporting information can be downloaded at: https://www.mdpi.com/article/10.3390/medicina62010146/s1.

## Abbreviations

The following abbreviations are used in this manuscript:

BF	Biceps femoris
BFlh	Biceps femoris long head
EMG	Electromyography
HSIs	Hamstring strain injuries
MVIC	Maximal voluntary isometric contraction
NHE	Nordic Hamstring exercise
RCH	Single-leg Roman chair-hold exercise
SENIAM	Surface Electromyography for Non-invasive Assessment of Muscles
