# Supplementary material for: Loaded Single-Leg Roman Chair Hold Preferentially Increases Biceps Femoris Activation, Whereas the Nordic Hamstring Exercise Emphasises Semitendinosus Activation in Professional Athletes
Source: Medicina (Kaunas). 2026 Jan 12;62(1):146. doi: 10.3390/medicina62010146 (PMC12843787; doi:10.3390/medicina62010146)
Supplement: Supplementary file 1 [file medicina-62-00146-s001.zip › medicina-4042567-supplementary.pdf]

Group	Gender	Age	weight	height_cm	BMI	Mean_Ove	Peak_Over	Mean_Ove	Peak_Over
1.00	1.00	20.00	61.00	168.00	21.61	70.85	88.90	59.32	75.78
1.00	2.00	29.00	106.00	194.00	28.16	54.00	77.75	13.79	19.19
1.00	1.00	19.00	56.00	168.00	19.84	40.00	49.79	46.29	51.89
1.00	1.00	17.00	65.00	175.00	21.22	43.76	52.76	41.02	46.43
1.00	2.00	24.00	96.00	185.00	28.05	69.55	80.38	63.43	68.84
1.00	2.00	23.00	78.00	185.00	22.79	80.17	104.08	48.23	55.23
1.00	2.00	19.00	100.00	193.00	26.85	41.28	43.97	104.32	128.32
1.00	1.00	18.00	68.00	174.00	22.46	60.74	75.66	32.79	37.07
1.00	1.00	27.00	58.00	166.00	21.05	72.01	82.15	44.04	46.54
1.00	1.00	25.00	74.00	176.00	23.89	59.67	59.99	47.41	55.55
1.00	2.00	22.00	85.00	179.00	26.53	45.33	55.32	54.58	69.04
1.00	2.00	17.00	72.00	176.00	23.24	72.98	87.30	62.64	72.55
1.00	1.00	21.00	79.00	174.00	26.09	23.79	28.28	31.24	38.05
1.00	1.00	26.00	53.00	164.00	19.71	43.67	51.22	60.45	64.74
1.00	2.00	17.00	75.00	175.00	24.49	58.10	69.66	45.40	58.19
1.00	2.00	27.00	83.00	184.00	24.52	87.54	100.87	54.65	65.49
1.00	1.00	25.00	73.00	172.00	24.68	43.49	55.03	26.47	30.44
1.00	2.00	22.00	86.00	180.00	26.54	57.00	68.52	24.54	27.91
2.00	1.00	19.00	57.00	160.00	22.27	36.29	51.88	42.21	51.88
2.00	2.00	19.00	85.00	180.00	26.23	39.80	52.24	42.43	53.75
2.00	2.00	26.00	96.00	190.00	26.59	50.79	88.58	53.86	72.93
2.00	2.00	25.00	85.00	180.00	26.23	40.49	56.90	61.08	84.19
2.00	1.00	17.00	76.00	177.00	24.26	38.86	63.24	33.56	51.08
2.00	1.00	37.00	66.00	173.00	22.05	30.21	43.23	49.94	72.23
2.00	2.00	23.00	73.00	173.00	24.39	56.69	55.49	47.63	53.98
2.00	2.00	22.00	78.00	178.00	24.62	36.45	59.18	48.10	65.87
2.00	2.00	25.00	82.00	188.00	23.20	41.97	46.56	80.08	121.10
2.00	2.00	23.00	88.00	185.00	25.71	54.73	60.92	45.39	47.23
2.00	1.00	23.00	63.00	178.00	19.88	36.64	51.46	23.90	31.85
2.00	1.00	27.00	63.00	174.00	20.81	40.35	60.40	62.80	99.63
2.00	1.00	23.00	62.00	170.00	21.45	55.26	68.97	54.37	72.20
2.00	1.00	17.00	63.00	156.00	25.89	57.92	64.89	60.98	73.58
2.00	2.00	18.00	89.00	198.00	22.70	51.09	77.15	62.66	78.91
2.00	1.00	19.00	63.00	168.00	22.32	51.97	79.10	69.65	98.51
2.00	2.00	18.00	76.00	186.00	21.97	63.57	83.61	49.85	58.78
2.00	2.00	26.00	79.00	183.00	23.59	54.28	77.09	69.17	89.46

Mean_RCH	Mean_RCH	Mean_RCH_3_BF	Peak_RCH_	Peak_RCH_	Peak_RCH_3_BF	Mean_RCH
50.79	73.02	88.73	62.32	94.59	109.78	43.24
42.19	46.87	72.95	76.76	61.64	94.85	9.69
43.68	41.57	34.75	59.19	49.45	40.74	34.84
20.99	50.96	59.33	28.19	61.74	68.36	15.28
55.00	66.95	86.70	61.85	79.26	100.04	51.74
69.40	81.13	89.97	101.52	99.33	111.39	35.65
23.54	45.40	54.90	22.49	48.78	60.63	80.61
37.85	45.82	98.55	46.91	66.06	114.01	23.29
43.56	74.60	97.86	47.27	89.00	110.19	21.47
41.83	61.57	75.60	48.66	58.72	72.60	27.14
41.76	49.46	44.77	57.96	55.30	52.70	55.23
54.08	75.41	89.45	60.37	89.68	111.86	44.88
18.09	22.55	30.73	20.89	27.82	36.13	20.70
27.92	39.10	64.00	34.65	48.67	70.33	34.35
56.37	56.32	61.62	61.70	72.80	74.49	38.00
67.18	82.58	112.85	73.48	98.83	130.31	44.95
23.55	37.29	69.64	27.01	48.89	89.19	18.61
44.61	47.53	78.86	44.82	57.26	103.49	19.51
36.29	36.29	36.29	51.88	51.88	51.88	42.21
39.80	39.80	39.80	52.24	52.24	52.24	42.43
50.79	50.79	50.79	88.58	88.58	88.58	53.86
40.49	40.49	40.49	56.90	56.90	56.90	61.08
38.86	38.86	38.86	63.24	63.24	63.24	33.56
30.21	30.21	30.21	43.23	43.23	43.23	49.94
56.69	56.69	56.69	55.49	55.49	55.49	47.63
36.45	36.45	36.45	59.18	59.18	59.18	48.10
41.97	41.97	41.97	46.56	46.56	46.56	80.08
54.73	54.73	54.73	60.92	60.92	60.92	45.39
36.64	36.64	36.64	51.46	51.46	51.46	23.90
40.35	40.35	40.35	60.40	60.40	60.40	62.80
55.26	55.26	55.26	68.97	68.97	68.97	54.37
57.92	57.92	57.92	64.89	64.89	64.89	60.98
51.09	51.09	51.09	77.15	77.15	77.15	62.66
51.97	51.97	51.97	79.10	79.10	79.10	69.65
63.57	63.57	63.57	83.61	83.61	83.61	49.85
54.28	54.28	54.28	77.09	77.09	77.09	69.17

Mean_RCH	Mean_RCH_3_ST		Peak_RCH_	Peak_RCH_	Peak_RCH_3_ST
58.87	75.85		55.45	75.03	96.85
12.08	19.60		13.96	16.05	27.57
51.92	52.12		39.37	56.11	60.18
47.60	60.18		17.48	52.18	69.64
59.39	79.17		51.02	67.75	87.76
45.88	63.17		36.99	52.41	76.30
103.36	128.98		92.59	132.90	159.46
27.68	47.41		26.88	32.47	51.86
46.83	63.83		21.79	50.95	66.87
45.64	69.45		30.28	57.57	78.79
52.38	56.14		76.02	66.59	64.50
67.11	75.92		46.95	80.11	90.58
27.36	45.66		23.54	34.61	55.99
53.53	93.47		43.34	59.76	91.13
46.54	51.66		45.44	60.52	68.61
50.75	68.25		57.73	60.16	78.59
19.25	41.55		17.48	21.07	52.77
21.50	32.60		18.76	24.52	40.44
42.21	42.21		51.88	51.88	51.88
42.43	42.43		53.75	53.75	53.75
53.86	53.86		72.93	72.93	72.93
61.08	61.08		84.19	84.19	84.19
33.56	33.56		51.08	51.08	51.08
49.94	49.94		72.23	72.23	72.23
47.63	47.63		53.98	53.98	53.98
48.10	48.10		65.87	65.87	65.87
80.08	80.08		121.10	121.10	121.10
45.39	45.39		47.23	47.23	47.23
23.90	23.90		31.85	31.85	31.85
62.80	62.80		99.63	99.63	99.63
54.37	54.37		72.20	72.20	72.20
60.98	60.98		73.58	73.58	73.58
62.66	62.66		78.91	78.91	78.91
69.65	69.65		98.51	98.51	98.51
49.85	49.85		58.78	58.78	58.78
69.17	69.17		89.46	89.46	89.46
